# Two eu*AGAMOUS* Genes Control C-Function in *Medicago truncatula*

**DOI:** 10.1371/journal.pone.0103770

**Published:** 2014-08-08

**Authors:** Joanna Serwatowska, Edelín Roque, Concepción Gómez-Mena, Gabriela D. Constantin, Jiangqi Wen, Kirankumar S. Mysore, Ole S. Lund, Elisabeth Johansen, José Pío Beltrán, Luis A. Cañas

**Affiliations:** 1 Instituto de Biología Molecular y Celular de Plantas (CSIC-UPV). Ciudad Politécnica de la Innovación, Valencia, Spain; 2 Plant Biology Division, The Samuel Roberts Noble Foundation, Ardmore, Oklahoma, United States of America; 3 Department of Plant Biology, Danish Institute of Agricultural Sciences, Frederiksberg C, Denmark; Universidad Miguel Hernández de Elche, Spain

## Abstract

C-function MADS-box transcription factors belong to the *AGAMOUS* (*AG*) lineage and specify both stamen and carpel identity and floral meristem determinacy. In core eudicots, the *AG* lineage is further divided into two branches, the eu*AG* and *PLE* lineages. Functional analyses across flowering plants strongly support the idea that duplicated *AG* lineage genes have different degrees of subfunctionalization of the C-function. The legume *Medicago truncatula* contains three C-lineage genes in its genome: two eu*AG* genes (*MtAGa* and *MtAGb*) and one *PLENA*-like gene (*MtSHP*). This species is therefore a good experimental system to study the effects of gene duplication within the *AG* subfamily. We have studied the respective functions of each eu*AG* genes in *M. truncatula* employing expression analyses and reverse genetic approaches. Our results show that the *M. truncatula* eu*AG*- and *PLENA*-like genes are an example of subfunctionalization as a result of a change in expression pattern. MtAGa and MtAGb are the only genes showing a full C-function activity, concomitant with their ancestral expression profile, early in the floral meristem, and in the third and fourth floral whorls during floral development. In contrast, *MtSHP* expression appears late during floral development suggesting it does not contribute significantly to the C-function. Furthermore, the redundant *MtAGa and MtAGb* paralogs have been retained which provides the overall dosage required to specify the C-function in *M. truncatula.*

## Introduction

Genetic regulation of flower development has been subject of study over the last decades, particularly in the model species *Arabidopsis thaliana* and *Antirrhinum majus*. These studies provided a general understanding of floral organ development in higher plants and led to the proposal of the ABCDE model, which postulates that floral organ identity in each whorl is defined by five functions named A, B, C, D and E (for review [1], [2], [3], [4]). In particular, C-function is required to promote stamen and carpel identity, to establish the determinate nature of the floral meristem [5] and also to repress A-function in the third and fourth whorls [6]. Cloning of ABCDE organ identity genes in *Arabidopsis* showed that most of them encode MADS-box transcription factors. Studies of MADS-box genes in higher eudicotyledoneous flowering plants show that they are key regulators of flower development.

In *Arabidopsis* and *Antirrhinum*, the C-function is essentially represented by a single gene respectively, *AGAMOUS* (*AG*) [5], [7] and *PLENA* (*PLE*) [8]. Additional C-function genes have been identified: *SHATTERPROOF1* (*SHP1*) and *SHATTERPROOF2* (*SHP2*) [9], [10] genes in *Arabidopsis* and *FARINELLI* (*FAR*) [11] in *Antirrhinum*.

Phylogenetic studies using a large data set of *AG*-like sequences show that *AG* and *PLE* actually represent paralogous lineages derived from a duplication in a common ancestor early in the history of the core eudicots. This duplication gave rise to the eu*AG* lineage which includes *AG* and *FAR,* and the *PLENA* lineage (*PLE*), where *SHP1*/*SHP2* and *PLENA* are placed [12], [13], [14], [15]. An even more ancient duplication occurred before the radiation of extant angiosperms, producing the C lineage (*AG* lineage) and the ovule-specific D lineage (*AGL11* lineage) [12], [15].

*AG, FAR* and *PLE* all display very similar expression patterns during the developing male and female reproductive organs [5], [8], [11]. Mutations of *AG* and *PLENA* in their respective species produce identical phenotypes characterized by developing flowers with petals in whorl 3 instead of stamens, and sepals in whorl 4 instead of carpels. In addition, the floral meristem is not determinate [7], [8]. However, *far* mutation only affects male reproductive organs, causing partial male sterility [11]. Meanwhile, *SHP* genes are only expressed in the ovules, in the developing pistil and show almost identical expression patterns in developing *Arabidopsis* fruit [16], [17], [18]. They function redundantly in style and stigma development, notably in the fusion of the carpel [19], and in seedpod shattering [10]. *SHP* and *FAR* genes do not contribute significantly to the C-function although FAR is expressed at early stages of flower development.

These studies showed a random evolutionary trajectory for gene functions after a duplication event. *AG* and *PLE*, different members of a duplicated gene pair, retained the primary homeotic functions in different lineages. Their respective orthologs have taken completely new roles in fruit dehiscence, as the case of *SHP1*/*SHP2* genes, or contribute redundantly in male reproductive development, as the case of *FAR*. Representatives of both eu*AG* and *PLE* lineages have been identified in different core eudicot species and several functional analyses are available for the paralogs from petunia, tomato, and *Nicotiana benthamiana* [20], [21], [22], [23].

In addition to the ancient duplication events observed in the *AGAMOUS* subfamily, more recent duplication events appear to have occurred in the eu*AG* lineage [12], [15], [24]. There have been reported two members of the eu*AG* lineage in some species, as for example *Populus trichocarpa* [25], *Cucumis sativus* [26], *Gerbera hybrida* [27], and in the legume species *Lotus japonicus* [28], *Pisum sativum* and *Medicago truncatula* (this study). However, in most cases, functional analyses are lacking. Both phylogenetic and functional data of gene lineages are very important to understand the evolution of gene families [12], [29].

*Medicago truncatula* (*Mt*) has three C-lineage genes: two eu*AG* genes (*MtAGa* and *MtAGb*) and one *PLENA*-like gene (*MtSHP*; [30]). To gain insight into the specific contribution of the eu*AG* and *PLE*-like paralogous genes in the control of the C-function, we compared the expression patterns of the three C-lineage genes during flower development. We have also characterized *MtAGa* and *MtAGb* loss-of-function mutants and plants where both genes have been simultaneously silenced. The particular capacity of these two genes to promote reproductive organ identity has been tested by ectopically expressing them in *Arabidopsis*. Our results indicate that the members of eu*AG* and *PLE* lineages in *M. truncatula* are subfunctionalized, where the C-function is only promoted by the eu*AG* paralogs. They largely overlap in function but the overall dosage of both gene products is critical to promote complete stamen and carpel identity and floral meristem determinacy in *M. truncatula*.

## Material and Methods

### Plant material and growth conditions

*Medicago truncatula* cv. Jemalong lines A17, SA1335 and R108, and *Arabidopsis thaliana* cv. Columbia plants were used in this study. Plants were grown in the greenhouse, at 22°C (day) and 18°C (night) with a 16 h light/8 h dark photoperiod, in soil:sand (3∶1) irrigated with Hoagland N^o^.1 solution supplemented with oligoelements [31].

The *mtaga* mutant allele (previously *matag-2*) was isolated in a previous screening [32] and homozygous plants were used in this study.

### Identification of Tnt1 insertion sites in *MtAGb* and co-segregation test

**Figure 1:**
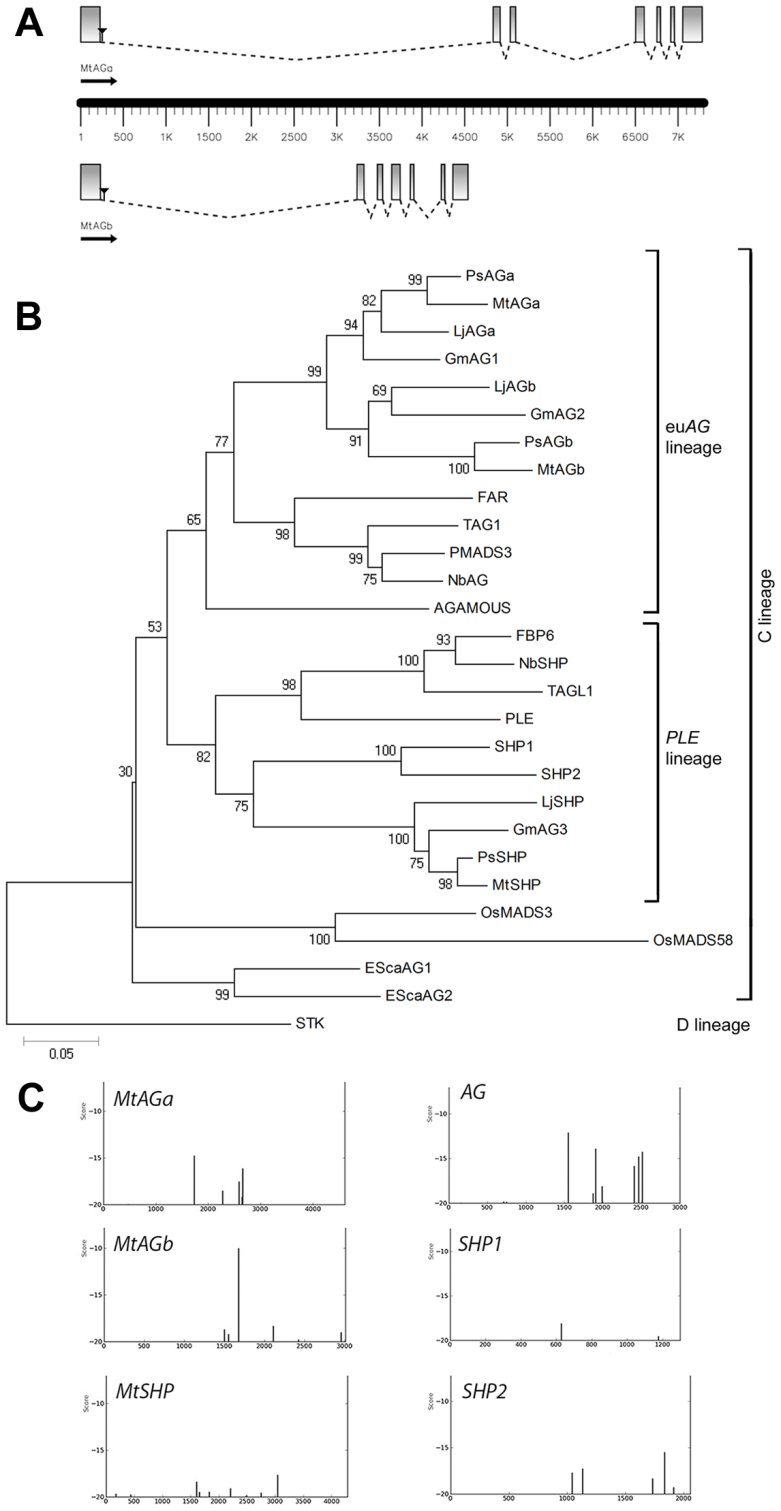
Two eu*AGAMOUS* genes in *Medicago truncatula*. (A) Gene structure of *MtAGa* and *MtAGb*. Coding sequences are represented as boxes and introns as dotted lines. Black triangles localize the position of the *Tnt1* insertions present in the mutant lines *mtaga* (NF13380) and *mtagb* (NF4908) used in this study. (B) Neighbor-Joining Tree of eu*AG* and *PLENA* homologs from a selection of diverse species. The numbers next to the nodes refer to bootstrap values from 10000 pseudo-replicates**.** (C) Distribution of putative LFY binding sites in the first intron of *MtAGa*, *MtAGb* and *MtSHP* genes as identified with the use of a position-specific scoring matrix using a cutoff value of −20.

The *M. truncatula* population used for the screening of mutants was described in detail [33], [34], [35], [36] (http://bioinfo4.noble.org/mutant/). The *mtagb* allele was identified by PCR screening of a segregating population of approximately 10,000 independent lines, using primers annealing to the *MtAGb* sequence (AGb-F, Table S2) in combination with primers annealing to the LTR borders of the *Tnt1* retroelement (Tnt1-F; Table S2 and Figure S3). We identified a line (NF4908) with an insertion of the retroelement located in the first intron at 277 bp of the starting codon (Figure 1A). The R1 plants were genotyped by PCR using the Tnt1-F primer in combination with the gene-specific primers MtAGb-F and MtAGb-Rgenomic (Table S2 and Figure S3B). Approximately 70% of the plants showed a mutant phenotype and co-segregated with the *Tnt1* insertion.

### Isolation and sequence analysis

*MtAGa and MtAGb* cDNAs were isolated from a library of *M. truncatula* A17 inflorescence apices [37], using the MADS-box fragment of the *M. truncatula PISTILLATA* gene as a probe. Sequence alignments and similarity comparisons of the inferred proteins were performed using Align and ClustalW tools [38]. The deduced amino acid sequences were aligned using ClustalW and further refined by hand. Genomic sequences search was performed using BLAST [39].

The second intron sequences were obtained by PCR using genomic DNA and the primers MtAGa-intron-F and MtAGa-intron-R for *MtAGa* and MtSHP-intron- F and MtSHP-intron-R for *MtSHP* (Table S2). To predict the probability of the presence of LFY binding sites in the intron sequences, we used the Morpheus webpage facility (http://biodev.cea.fr/morpheus/Default.aspx) with the LFY matrix. The score tool permits localization of the best transcription factor binding sites in DNA fragments.

### Phylogenetic tree

The phylogenetic tree was inferred by Neighbor-Joining using Poisson-Corrected amino acid distances. Reliability of internal nodes was assessed using bootstrap with 10000 pseudo-replicates. Tree inference was conducted using *MEGA* version 4 [40]. The data set comprised 28 previously reported C- and D-class genes obtained from GenBank and the two new sequences that we isolated (*MtAGa* and *MtAGb*). The tree was rooted using the *Arabidopsis* D-class gene *SEEDSTICK* (*STK*) sequence. All sequences used in this analysis, with their respective species and accession numbers, are included in Table S1.

### Southern blot hybridization

Plant genomic DNA was extracted from leaves as described by [41]. Ten micrograms of DNA were digested with restriction enzymes, separated on 0.7% Tris-borate EDTA 1X agarose gels overnight at 1V/cm and transferred to a nylon membrane. Southern blot hybridization was performed by standard methods at 65°C. A 241 bp fragment of *MtAGa* cDNA (positions 572-813) and a 215 bp fragment of the *MtAGb* cDNA (positions 558-773) were amplified with the specific primers MtAGadir, MtAGarev, MtAGbdir and MtAGbrev1 (Table S2) and used as probes.

### Expression analyses

Total RNA was isolated from frozen plant material using the RNeasy Plant mini Kit (Qiagen, Germany) according to the manufacturer's instructions.

For Northern blot analysis total RNA (15 µg) from frozen leaves, roots, stems, flowers and pollinated ovaries (young fruits) was used. RNA electrophoresis was carried out in formaldehyde-agarose gels, transferred to Hybond N^+^ membranes (Amersham Biosciences, USA), and hybridized with ^32^P-labeled probes under standard conditions. The probes were generated from the same gene fragments used for Southern blot analysis.

For Real Time RT-PCR analysis total RNA was treated with *rDNase*I of the *DNase* Treatment and Removal Kit (Ambion, Life Technologies, USA). For first-strand synthesis, total RNA (1 µg) was reverse-transcribed in a 20 µl reaction mixture using the PrimerScript 1^st^ strand cDNA Synthesis Kit (Takara, Japan). One microliter of RT reaction was used for a Real Time RT-PCR analysis with 300 nM of each primer mixed with the Power SYBR Green PCR Master Mix (Applied Biosystems) according to the manufacturer's instructions. The reaction was carried out into 96 well-optical reaction plates using an ABI PRISM 7500 Sequence Detection System and appropriate software (Applied Biosystems). The relative levels were determined by the 2 ^–ΔΔ*Ct*^ Method [42]. To normalize the variance among samples, *Secret Agent* (*O-linked N-acetyl glucosamine transferase*: TC77416; [43]) was used as an endogenous control. All reactions were performed by triplicate using a biological replicate for each sample. Primers were designed using PRIMER EXPRESS software (Applied Biosystems, USA) using default parameters and are listed in Table S2.

RNA *in situ* hybridization was performed on 8 µM paraffin sections of *M. truncatula* inflorescences as described by [16], using digoxigenin-labelled probes. The RNA sense and antisense probes were generated from the same gene fragments used for Southern blot analysis. Both fragments were cloned into the pGEM T-easy vector (Promega, USA) and the probes were synthesized using SP6 or T7 RNA polymerases.

### Virus Induced Gene Silencing in *Medicago truncatula*

pCAPE1 and pCAPE2 derivatives were used as vectors for gene silencing [44]. Two DNA fragments from the 3′ region of the *MtAGa* (310 bp) and *MtAGb* (338 bp) genes were obtained by PCR using primers (MtAGaVIGSdir, MtAGbVIGSdir, MtAGaVIGS2rev, MtAGbVIGS2rev) that added restriction sites to both ends of the fragments (Table S2). The amplicons were cloned into pGEM T-easy (Promega, USA), digested using the appropriated restriction enzymes and cloned into a similarly digested pCAPE2 vector [44]. The resulting plasmid (pCAPE2-*MtAGab*) was confirmed by sequencing before being introduced into *Agrobacterium tumefaciens* strain C58/pMP90. *Agrobacterium* inoculation of *M. truncatula* leaves was performed as described by [44].

### Generation of transgenic RNAi plants

Transformation of *M. truncatula* R108 was performed as described previously [33]. The 35S::RNAi-*MtAG* construct was performed using a 215 bp fragment from *MtAGb* (positions 557–772 from the ATG codon), amplified using primers MtAGb-RNAiD and MtAGb-RNAiR (Table S2) that incorporate two restriction sites that are used for cloning into the pHANNIBAL vector [45].

### *Arabidopsis* transformation

*MtAGa* and *MtAGb* cDNA fragments were amplified using AGaSBXdir, AGaSBXrev, AGbSBXdir and AGbSBXrev primers (Table S2) and cloned into the pBINJIT60 vector [46], a pBIN19 derivative (Clontech, Palo Alto, CA, USA), which placed gene transcription under the control of a tandem repeat of the CaMV 35S promoter. *Arabidopsis* plants were transformed by floral dipping according to standard procedures [47] and selected on kanamycin.

### Photography, microscopy and cryo-SEM

Light photographs were made with a stereomicroscope Leica MZ16F attached to a DFC300 FX camera (Leica Microsystems, Germany).

After *in situ* hybridizations, sections were observed and photographed with a Nikon Eclipse E-600 microscope equipped with a digital camera.

For cryo-SEM, samples were frozen in slush nitrogen and attached to the specimen holder of a CryoTrans 1500 Cryo-Preparation System (Oxford Instruments, UK) interfaced with a JEOL JSM-5410 scanning electron microscope. The samples were then transferred from cryostage to the microscope sample stage, where the condensed surface water was sublimed by controlled warming to -85°C. Afterwards, the sample was transferred again to the cryostage in order to gold coat it by sputtering. Finally the sample was put back on the microscope sample stage to be viewed at an accelerating voltage of 15 KeV.

## Results

### Identification of two eu*AGAMOUS* genes in *Medicago truncatula*

To identify MADS-box genes involved in flower development, we screened a cDNA library of *M. truncatula* floral apices using a set of MADS-box fragments from different species as a probe [48], [49]. Among the isolated clones, three corresponded to full-length sequences that present significant similarity to genes of the *AGAMOUS* subfamily. One of these clones presents high sequence similarity with the *Arabidopsis SHP1* and *SHP2* genes [17], [18], [50] and with the *Antirrhinum PLENA* gene (*PLE*; [8]) and corresponds to the *MtSHP* gene [30]. The other two clones isolated present high sequence similarity with the C-lineage genes *AGAMOUS* of *Arabidopsis* [5] and *FARINELLI* of *Antirrhinum* [11] and have been named *MtAGa* and *MtAGb*. The *MtAGa* clone is 1208 bp long, with an ORF of 780 bp and a deduced protein of 260 amino acids. The *MtAGb* clone is 1099 bp long, with an ORF of 732 bp and the deduced protein has 244 amino acids. MtAGa shows 81% amino acid identity with MtAGb, being more similar in the N-terminal domains (98% in the MADS domain, 76% in the I region and 80% in the K domain) than in the C-terminal region (68% of identity). MtAGa and MtAGb show respectively 71 and 68% amino acid identity with the eu*AG* clade protein FAR and either one 67% amino acid identity with AG (Figure S1).

The *MtAGb* genomic sequence was found in two BAC clones: mth2-30e7 (GenBank AC137837.4) and mth2-76i7 (GenBank AC153460.24). This gene is organized in seven exons and six introns (GenBank KJ470634; Figure 1A). The genomic sequence of the *MtAGa* gene was not available in the databases and was obtained in this study (GenBank KJ470633). *MtAGa* gene is also organized in seven exons and six introns (Figure 1A). Both genes are present as single copy in the genome as confirmed by Southern blot analysis (Figure S2A). Genomic sequences from *MtAGa* and *MtAGb* genes were used as BLASTN queries, and displayed using the Chromosome Visualization Tool (CViT, http://www.medicagohapmap.org/tools/blastform). We found that *MtAGb* gene is located on chromosome 8 (Figure S2B). However, no location was obtained for the *MtAGa* gene.

Phylogenetic analyses showed that *MtAGa* and *MtAGb* are relatively recent paralogs within the *euAG* subclade (Figure 1B). Other model legumes such as *Pisum*, *Lotus* and *Glycine* used in this analyses, also displayed two paralogs in this subclade. In most of the studied plants, the eu*AG* subclade is represented by one single gene, such as *AG* for *Arabidopsis*, *FAR* for *Anthirrinum* or *TAG1* for tomato.

Genes from the eu*AG*- and *PLE*-subclades present a well conserved gene structure and characteristic large introns essential for their correct expression pattern [51], [52]. In *M. truncatula*, both *MtAG* genes show a large first intron (4607 bp in *MtAGa* and 3007 bp in *MtAGb*). We searched in these sequences for the presence of putative LFY binding sites that are characteristic of C-function genes. We included in this study the *MtSHP* gene and then we have sequenced its first intron of 4281 bp (GenBank KJ470635). We used a bioinformatics tool available at the Morpheus webpage facility (http://biodev.cea.fr/morpheus/Default.aspx) which examines the distribution of individual LFY binding sites. The presence or absence of LFY binding sites identified by this approach appears to be helpful in predicting to what extent a C-clade gene acts as a true C-function gene [53]. Our results show different binding site landscapes for the three genes analysed (Figure 1C). In the case of the *MtAGb* first intron, there is a main single binding site of very high score similar to the profile found for the *PLE* gene [53]. This site corresponds to two fully intact CCAAT-boxes. In the first intron of *MtAGa*, lower affinity sites are present but the binding seems to be compensated through the action of several nearby sites as reported for the sites present in the second intron of *AG* (Figure 1C). The prediction of binding sites for LFY in the first intron of *MtSHP* gave only low score values (Figure 1C) indicating a low probability to be regulated by LFY at early stages of floral development.

Globally, these data suggest that the duplicated eu*AG* genes *MtAGa* and *MtAGb* might contribute to C-function specification in *M. truncatula* and play a role during early stages of flower development.

### Expression patterns comparison of three *M. truncatula* C-lineage genes during floral development

**Figure 2:**
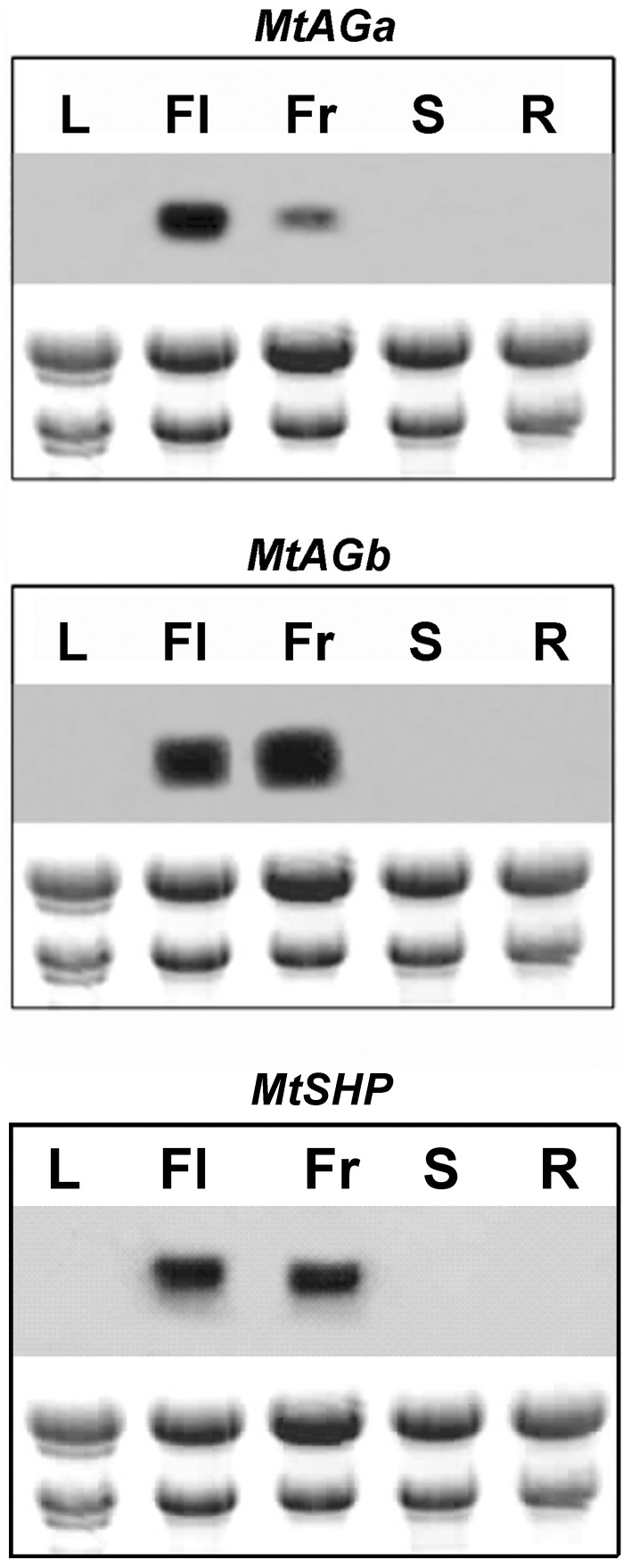
Expression patterns of *MtAGa, MtAGb* and *MtSHP* genes in various plant tissues of *M. truncatula*. Northern blot analyses were performed using total RNA prepared from leaves (L), flowers (Fl), young fruits (Fr), stems (S) and roots (R).

**Figure 3:**
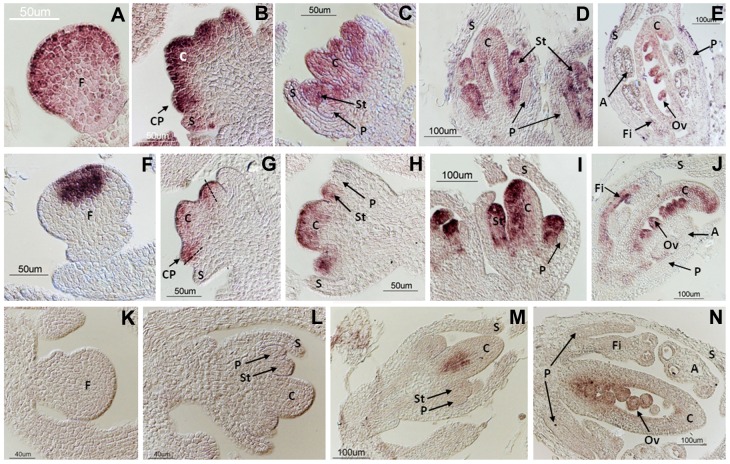
Expression pattern of *MtAGa*, *MtAGb* and *MtSHP* genes during early floral development. *In situ* localization of *MtAGa* (A-E), *MtAGb* (F-J) and *MtSHP* (K-N) transcripts in *M. truncatula* wild-type flower buds. Developmental stages were defined according to [74]. *MtAGa* transcripts are localized in the whole floral meristem at stages 2 (A) and 4 (B). At stage 5 (C) *MtAGa* mRNA is detected in stamen and carpel primordia. At stage 7 (D-E) expression locates in stamens, carpel and the developing ovules. *MtAGb* transcripts are localized in the center of the floral meristem at stage 2 (F). At stage 4 (G) expression locates in the carpel primordia and the half of the common primordia that will give rise to the stamens. At stage 5 (H) *MtAGb* mRNA is detected in stamen and carpel primordia. At stage 7 (I-J) *MtAGb* mRNA is detected in stamens, carpel and developing ovules. *MtSHP* transcripts are not detected at stages 2 (K) and 5 (L). At stage 6 *MtSHP* transcripts are localized in the inner cells of the developing carpel (M). At stage 7 (N) expression can be detected in the developing ovules. F: floral meristem; S: sepal; C: carpel; CP: common primordia; St: stamen; P: petal; Fi: filament; A: anther; Ov: ovule.

The expression patterns of *MtAGa*, *MtAGb* and *MtSHP* were analysed by Northern blot in different plant tissues and the three genes are exclusively expressed in floral and young fruit tissues (Figure 2). We performed detailed *in situ* hybridization experiments to show the distribution of *MtAGa*, *MtAGb* and *MtSHP* mRNAs during floral development (Figure 3). Both *MtAGa* and *MtAGb* transcripts began to accumulate at stage 2 of flower development. At this stage, *MtAGb* accumulates in the centre of the floral primordia while *MtAGa* signal was detected throughout the floral meristem (Figure 3F and 3A). At stage 4, *MtAGb* transcript was located in the region of the common primordia that will give rise to the stamens and also in the central part of the floral apex where the carpel is developing (Figure 3G). However, *MtAGa* expression is still observed on the whole floral meristem, including petal and sepal primordia (Figure 3B). From stage 5, expression of both paralogs was distributed uniformly in whorls 3 and 4 (Figure 3C-E and 3H-J), although hybridization signal for *MtAGb* was stronger on the abaxial region of the carpel. In later stages, expression of both transcripts was observed in the developing ovules, in the distal region of the carpel and on the filament of the anthers (Figure 3E and 3J). In contrast, *MtSHP* mRNA began to accumulate late on flower development and can be detected at stage 6 in the inner cells of the developing carpel (Figure 3M). Since late stage 7, *MtSHP* expression is exclusively detected in the ovules (Figure 3N).

We conclude that the expression of the two *M. truncatula* eu*AG* genes remained largely restricted to the developing male and female organs from early stages and throughout flower development. *MtAGa* and *MtAGb* showed nearly identical spatial expression patterns from stage 4 to late developmental stages. Differences were mainly observed at early stages, where *MtAGa* expression appeared to be wider spread in the floral meristem than *MtAGb*. Essentially, the duplicated *MtAGa* and *MtAGb* genes showed similar spatial and temporal expression patterns to those described for C-function genes from the eu*AG* subclade such as *AG* and *PLE*. In contrast, *MtSHP* was only expressed in the developing carpel and in the ovules, similar to the pattern described for *Arabidopsis SHP* genes that do not significantly contribute to C-function.

### *MtAGa* and *MtAGb* loss-of-function analyses

**Figure 4:**
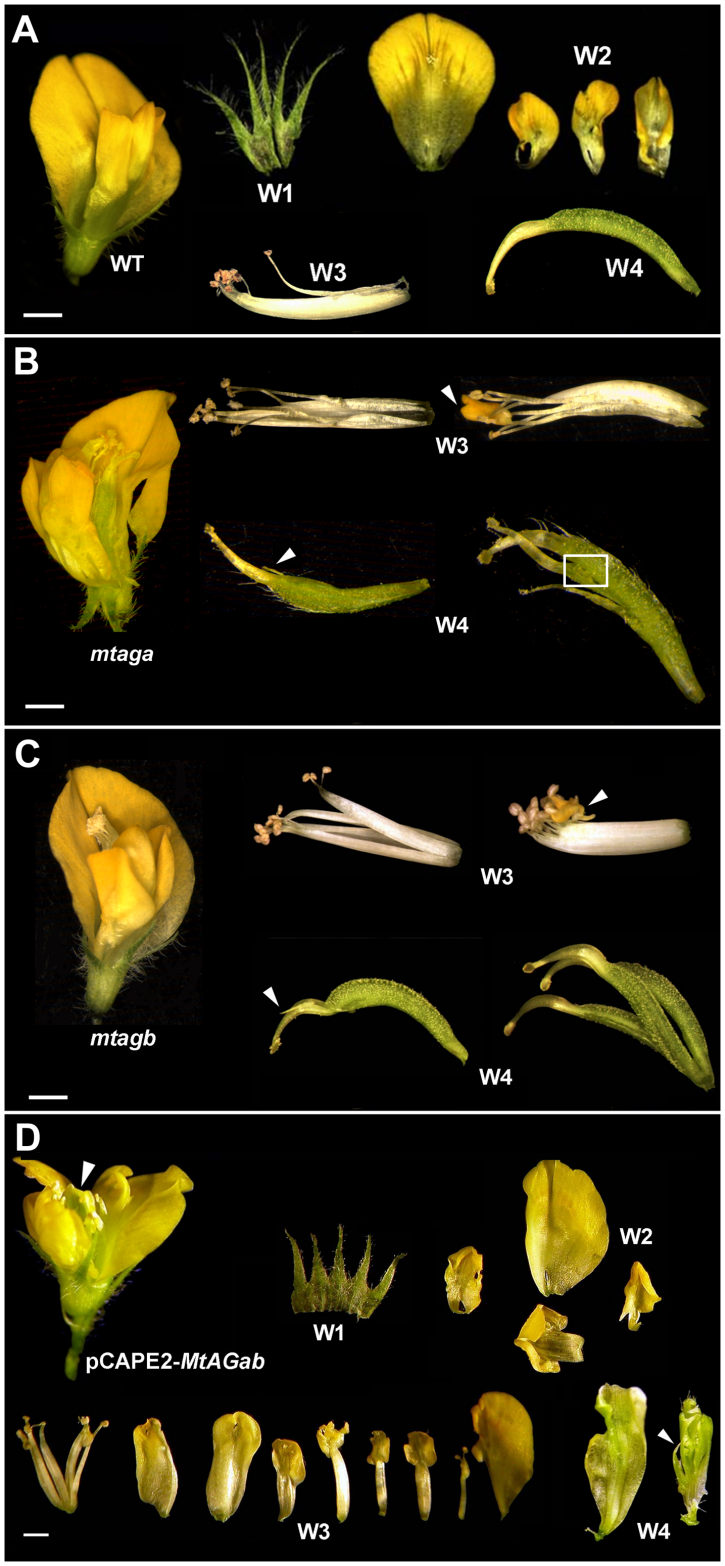
Floral phenotypes of *M. truncatula mtagb* and *mtaga* mutants and MtAGab-VIGS silenced plants. (A) Dissected wild-type *M. truncatula* flower showing the four floral whorls (W1 to W4). Floral phenotypes are similar in *mtaga.* (B) and *mtagb* (C) mutants. Mutant flowers (left) were opened to show the inner whorls. In W3 the staminal tubes are unfused and occasionally petaloid structures appear (arrow) replacing the anthers. Carpels in W4 present stigmatic protuberances (arrow) or multiple unfused carpels and exposed ovules (boxed). (D) *MtAGab-*VIGS flower with severe homeotic transformation of stamens into petals and carpels into sepaloid structures (arrows). Bars indicate 1 mm.

To investigate the specific contribution of *MtAGa* and *MtAGb* genes in the *M. truncatula* floral development, we looked for retrotransposon insertion mutants [32]. These mutants were available for both genes, each containing a single *Tnt1* element inserted in the N-terminal part of the gene (Figure 1A). *mtagb Tnt1* insertion was identified as described in the experimental procedures and it was located in the first intron at 277 bp from the start codon (Figure 1A). *mtaga* (previously *mtag-2*), was isolated as a case study to demonstrate the utility of the reverse genetic platform in the model legume *Medicago truncatula* [32]. Using quantitative RT-PCR we have confirmed that *MtAGa* or *MtAGb* transcripts were nearly undetectable in the corresponding homozygous mutant plants without affecting the expression levels of the corresponding *MtAG* paralog (Figure S4). *mtaga and mtagb* mutants exhibit very similar floral phenotypes: flowers are wild-type in appearance, showing only mild developmental defects on the third and fourth-whorl organs (Figure 4). Incomplete fusion of the staminal tube was observed and occasionally, some petaloid prolongation in the anther tip (Figure 4B and 4C). Whorl 4 is frequently composed by multiple carpels (2-3) or by modified carpels with stigmatic protuberances and exposed ovules (Figure 4B and 4C). Flowers were generally sterile, although occasionally a few seeds were produced.

To investigate the effect of the combined loss-of-function of *MtAGa* and *MtAGb*, we performed virus induced gene silencing (VIGS) experiments. We used a VIGS vector based on PEBV (*Pea early browning virus*) initially developed to induce gene silencing in *Pisum* [44]. The silencing efficiency of this vector is lower in *M. truncatula* cultivars than in *P. sativum* [54]. We generated the pCAPE2-*MtAGab* construct to simultaneously silence both genes (see Materials and Methods). Twenty one plants were inoculated with the pCAPE2-*MtAGab* construct and we analysed the phenotype of 150 flowers. 10% of the MtAGab-VIGS flowers showed homeotic transformations in whorls 3 or 4. Only 1% of the MtAGab-VIGS flowers showed a near complete loss-of-C-function phenotype (Figure 4 D). In whorl 3 some anthers are completely converted into petal-like organs, and in the fourth whorl, sepal-like structures arise instead of carpels (Figure 4 D). These flowers also showed indeterminacy of the floral meristem revealed by the presence of multiple petaloid or sepaloid concentric structures in the centre (Figure 4D, arrows). This phenotype suggests that both *MtAG* genes were strongly down-regulated but, because of floral tissue limitation, we could not determine the expression levels of the targeted genes.

In parallel, we also developed transgenic *M. truncatula* lines in which *MtAG* genes were down regulated by RNA interference (RNAi) and three independent lines were obtained. Only one of these lines (line 5.7) showed floral phenotypes similar to those of single *mtag* mutants (Figure S5). These flowers showed clear morphological aberrations consisting of mild homeotic transformation of stamens to petals and multiple carpels with exposed ovules. We analyzed the expression of both *MtAGa* and *MtAGb* genes in the three RNAi lines. These lines showed a different degree of silencing for each targeted gene (Figure S5J). In silenced line 1.3, we found *MtAGb* levels considerably reduced (∼15% of wild-type level), while *MtAGa* levels are somewhat decreased (∼90% of wild-type level). Strong silencing of one paralogous gene did not produce homeotic floral defects. However, floral defects in line 5.7 correlate with simultaneous downregulation of both genes (∼40% of wild-type level) (Figure S5J).

### *MtAGa* and *MtAGb* gain-of-function analyses

**Figure 5:**
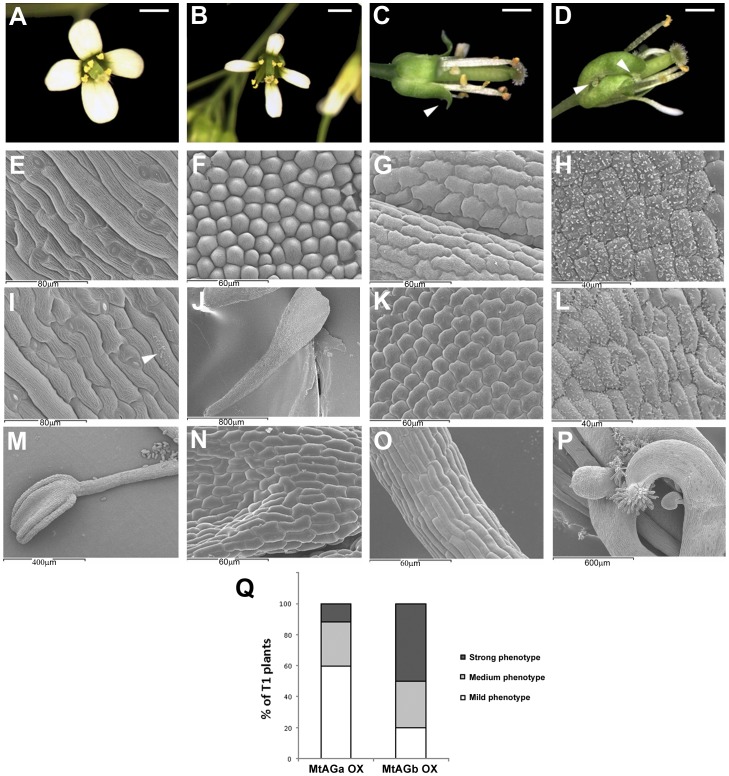
Overexpression of *MtAGa* and *MtAGb* in *Arabidopsis thaliana*: floral phenotypes. Wild-type *Arabidopsis* flower (A). Floral phenotypes observed in *Arabidopsis* plants overexpressing *MtAGa* or *MtAGb* genes have been classified into mild (B), medium (C) and strong (D). Scanning electron micrographs showing the characteristic cellular types of wild-type sepals (E), petals (F), stamens (G) and carpels (H). Scanning electron micrographs of sepals from overexpression lines with mild phenotype (I) showing wax accumulation (arrow). Narrow petals from overexpressing lines (J) showing staminoid cellular types (K). Wax accumulation in sepals (L) of plants with medium and strong phenotypes. Near complete homeotic conversion of petal into stamen (M) showing characteristic cellular types from anther (N) and filament (O). Flower with strong phenotype showing ectopic ovules and stigmatic tissue (P) in the first whorl. Relative proportion of phenotypes observed in plants overexpressing *MtAGa* or *MtAGb* genes (Q). Bars indicate: 1mm in A, B, C, and D.

Ectopic expression of *AG* results in the homeotic conversion of sepals and petals into carpels and stamens, respectively [55], [56]. Other reported phenotypes include early flowering and curling of leaves. To investigate whether MtAGa and MtAGb proteins differ in their ability to induce reproductive organ fate we overexpressed these two genes in *Arabidopsis*. The coding sequence of these genes was located under the control of the CaMV 35S constitutive promoter. We analysed 75 independent primary transgenic (T1) lines for every construct (*35S*::*MtAGa* and *35S*::*MtAGb* plants) and found plants with different floral phenotypes that we classified as mild, medium and strong phenotype (Figure 5B-D). Transgenic plants with mild phenotype showed wax accumulations on the surface of the sepals (Figure 5I) that are characteristic of the carpel (Figure 5H). The petals are narrow and asymmetric (Figure 5B and 5J) with partial homeotic conversions to stamens (Figure 5K). Flowers with medium phenotypes showed curved sepals (Figure 5C) with abundant accumulation of wax (Figure 5L). T1 plants with medium and strong phenotypes showed near complete homeotic transformations of sepals to stamens (Figure 5M and 5N). Finally, flowers with strong phenotype showed thickened and curved sepals (Figure 5D) that contain carpelloid structures with ectopic ovules and stigmatic papillae (Figure 5P). Although these three phenotypical classes were observed for both constructs, we notice a higher proportion of plants with medium/strong phenotypes (80.3%) in the *MtAGb* overexpressing lines (Figure 5Q). In contrast, 59.5% of *MtAGa* overexpressing lines showed mild homeotic transformations.

In summary, constitutive expression of *MtAGa* and *MtAGb* in *Arabidopsis* are able to cause equivalent homeotic alterations in the flowers that are essentially identical to those caused by constitutive expression of other eu*AG* genes [56]. However MtAGb protein seems to be more efficient than MtAGa to induce reproductive organ fate when overexpressed in a heterologous system.

## Discussion

The model legume *Medicago truncatula* harbors two members of the eu*AG* sub-clade (*MtAGa* and *MtAGb*) and one member of *PLENA* (*MtSHP*) [30]. In this study, we report the functional characterization of the eu*AG* homologs from this species to achieve a more thorough understanding of the evolution of the *AGAMOUS* subfamily in core eudicots.

### Two euAG genes in Medicago truncatula

The finding of two eu*AG* duplicated genes (*MtAGa* and *MtAGb*) in *M. truncatula*, as well as in other legume species (see Figure 1B) suggests that each pair of paralogs arose by a duplication event prior to the speciation of legumes. They could have been originated during the whole-genome duplication (WGD) event that predated speciation of *M. truncatula* and other legumes around 50-60 Mya [57], [58]. Gene duplications allowed the emergence of critical changes in several MADS-box gene lineages that control floral organ identity. In *Medicago*, B-function genes are also duplicated: *MtAP3-like* genes were originated by an ancient duplication concomitant with the base of the core eudicot radiation [49], whereas the two duplicated *PI-*like genes were probably originated during the WGD event that also originated the two *MtAG* genes ([48]; Roque *et al*. unpublished). The changes of these genes after duplication may have played an important role in the evolution of floral morphology and ontogeny in legumes.

The presence of two eu*AG* genes is not only found in legumes. Previous phylogenetic studies have revealed the existence of two members of the eu*AG*-lineage in several core eudicots [12]. This is the case of the paralogous *CUM1* and *CUM2* from *Cucumis sativus*, *PTAG1* and *PTAG2* from *Populus trichocarpa*, and *GAGA1* and *GAGA2* from *Gerbera hybrida*. These studies are mainly focused on the analyses of their expression patterns and limited functional information is available. Recently, phylogenetic analyses using AG protein sequences from several Asteraceae species, have revealed that some of them, harbor two or three members within the eu*AG*-lineage [24].

MtAGa and MtAGb proteins conserve characteristic domains of the C-lineage members, as the presence of an N-terminal extension preceding the MADS domain [59]. The length of this sequence in MtAG proteins falls within the range previously described for other members of this clade (from 13 to 52 amino acids) [59] (Figure S1). However, regarding to DNA sequence, both genes possess six introns rather than the typical eight of the other *AG* homologs [7], [8], [11], [25], [60], [61]. This genomic organization is observed in both angiosperm and gymnosperm genes suggesting that the presence of eight introns is likely to be primitive in *AG* subfamily [12]. The genomic organization of *M. truncatula* eu*AG* genes could have taken place during the WGD event that predates legume speciation followed by gene rearrangement.

Within the highly similar gene structure of *AGAMOUS* genes, it has been shown that the first and second introns are essential for their correct expression [24]. Among the identified enhancers, the transcription factor *LFY* is essential for *AG* activation and binds to *cis*-elements located in the second intron [52], [62]. The presence of LFY-binding sites has been reported to be critical to establish early expression of C-function genes during flower development [63]. We identified high affinity binding sites for LFY in the first intron of both *MtAGa* and *MtAGb*. Although the binding site landscapes observed for each eu*AG*-paralogous gene are different, experimental analyses have demonstrated that both types of distribution of LFY binding sites are functionally relevant [53]. In contrast, in our analyses *MtSHP* was not found to be a likely LFY target and accordingly, *MtSHP* expression is exclusively detected in the ovules at later stages of floral development. Similarly, *SHP* or *STK*, which play late roles in fruit and ovule development, were not found to be LFY targets in ChIP-seq experiments [53]. Together, our analyses suggest that both *MtAG* genes have the same probability to be directly activated by LFY, conferring organ identity on floral meristems that arise after the transition to reproductive development.

### Two eu*AG* genes control C-function in *Medicago truncatula*

A typical C-function knock-out phenotype is described as the conversion of stamens to petals, and a flower in place of the fourth whorl, which starts with a whorl of sepals [24]. 1% of MtAGab-VIGS flowers showed a near complete loss-of-C-function phenotype. Stamens were replaced by petals or petal-like organs and the central carpel was replaced by multiple petaloid or sepaloid concentric structures. MtAGab-VIGS plants still showed some reproductive structures probably due to the fact that these lines still produce some *MtAGa* and *MtAGb* transcripts. It would be desirable to measure the *MtAG* mRNAs levels in MtAGab-VIGS flowers to validate this hypothesis, but sufficient plant material was not available. However, using stable *MtAG*::RNAi lines we were able to correlate the amounts of *MtAG* gene products with partial loss-of-C-function phenotypes. Only simultaneous down-regulation of both genes (below 40% of the wild-type level) produces mild aberrations in stamen and carpel development. These results support the quantitative model of AG activity, which suggests that the different *AG* functions vary with respect to the amount of gene product required [47]. The floral determinacy requires the higher levels of AG, while low levels of AG are sufficient for carpel and stamen specification [51]. According to this model, only a strong reduction of both *MtAG* transcripts could explain the indeterminacy of the floral meristem in MtAGab-VIGS flowers. In each single *mtaga* and *mtagb* mutants the amount of the remaining paralog is sufficient to confer almost all the C-function. However, the discrete mutant phenotypes of these plants suggest that a single *MtAG* gene is not sufficient to maintain the overall dosage of these subcomponents of protein complexes which are needed to specify the C-function in *M. truncatula.*

The spatial and temporal expression profile of *AGAMOUS* subfamily genes is closely consistent with their conserved function and subfunctionalization [24]. The expression of *AG* lineage genes early in the floral meristem, and in the third and fourth floral whorls is probably the ancestral expression profile of these genes [24]. An overlapping conserved expression pattern for both eu*AG* and *PLE*-like genes is observed in *Petunia*, *N. benthamiana* and *Antirrhinum*, at early floral developmental stages. This expression profile correlates with their functional redundancy to promote the C-function of these genes in the cited species [4], [11], [20]. In *A. thaliana*, the only C-function factor is *AG*, because, although the PLE-like proteins (SHP proteins) fully maintain a similar potential activity, they are not physically present in the meristem and primordia cells at the appropriate time. Their functions are rather restricted to regulate specific tissue types after carpel identity has been established. It remains possible that *SHP* genes redundantly regulate carpel identity with *AG* because they are expressed in early carpel primordia. The Arabidopsis *AG* and *SHP* genes are a clear example of subfunctionalization as a result of a change in expression pattern [64]. *M. truncatula* could be considered an illustrative example, where the expression pattern of the members of eu*AG* or *PLENA* lineages would be very well correlated with their function. The two eu*AG* genes (*MtAGa* and *MtAGb*) are expressed early during floral development, having a critical role in the specification of reproductive organs and floral determinacy. *MtSHP* expression appears late during floral development after all organs have been specified, suggesting it does not contribute significantly to the different components of the C-function (stamen and carpel identity, and determinacy). A role for this gene in fruit morphology and valve margin lignification has been suggested [30]. However, we cannot exclude a function for *MtSHP* in ovule development, associated with its expression in this tissue during floral development.

It seems that changes in coding sequences are not the main cause of the functional divergence between the majority of the eu*AG* and *PLENA*-like members. In most cases, the ectopic expression of each sub-clade member gave similar results [4], [23], [55], [65], [66], [67]. This demonstrates that both types of proteins have conserved their biochemical interactions. However, an exception to the rule has been observed, for example, in *Antirrhinum*, where the presence of an additional glutamine in the K3 helix of FAR protein, causes its inability to induce carpel identity in the first whorl [68], [69]. These data suggest that the functional diversification of *Antirrhinum* C-lineage genes largely relies on changes in the protein ability to interact rather than on changes in their expression patterns. The ectopic expression of *M. truncatula* eu*AG*- and *PLENA*-like genes (*MtAGa*, *MtAGb* and *MtSHP;* this work; [20]) in *Arabidopsis* was able to cause homeotic alterations in the flowers that are essentially identical to those caused by the constitutive expression of other eu*AG-* and *PLENA*-like genes. However, we noticed that although there are not qualitative differences in the floral phenotypes between *35S*::*MtAGa* and *35S:*:*MtAGb* transgenic plants, they differ quantitatively, indicating that MtAGb protein could be more active in *Arabidopsi*s than MtAGa.

Overall, our results show that *MtAG* genes could have the same probability to be regulated by LFY at early stages of floral development, while the prediction of binding sites for LFY in the first intron of *MtSHP* indicated a low probability to be regulated by LFY. Both *MtAG* genes showed nearly identical spatial expression patterns, which are similar to those described for C-function genes from the *euAG* subclade such as *AG* and *PLE*. In contrast, *MtSHP* is exclusively detected in the ovules at late stages of floral development. It is not physically present in the meristem and primordia cells at the appropriate time to participate in the C-function. Therefore, in *M. truncatula*, the C-function is redundantly encoded by the two eu*AG*-genes. Finally, the overexpression of *MtAGa* or *MtAGb* in *Arabidopsis*, gave similar results to those observed for the ectopic expression of other eu*AG*-genes. *M. truncatula* eu*AG*- and *PLENA*-like paralogs could have subfunctionalized concomitantly with the differential spatial and temporal expression pattern of the ancestral gene lineage. Furthermore, *MtAG* paralogs are maintained in the genome consistent with the gene balance hypothesis, which predicts that the fate of duplicated genes largely depends on the maintenance of the stoichiometric balance among members of the macromolecular complex [70], [71], [72], [73].

*AG* subfamily has been shaped by a complex history of gene duplications leading to various degrees of redundancy and/or subfunctionalization of the C-function in individual gene copies. Despite these differences, the control of the development of reproductive organs is strongly conserved across eudicots. We provide insight on the evolution of the *AG* subfamily in a new core eudicot species, highlighting the dynamic nature of functional evolution following gene duplication in this MADS-box gene subfamily.

## Supporting Information

Figure S1 **Sequence comparison of MtAGa, MtAGb and related MADS-box proteins.** Identical amino acid residues are shaded in black, and similar amino acid residues in grey. The MADS and K domains, and I and C-terminal regions are marked with arrows. The AG motifs I and II are underlined with continuous and discontinuous lines, respectively. A dotted line before the MADS-box indicates the N-terminal sequence characteristic to some C-lineage genes.
(TIF)

Figure S2 **Southern blot analysis of** ***MtAGa*** **and** ***MtAGb*** **genes and localization of the** ***MtAGb*** **gene on the** ***M. truncatula*** **physical map.** (A) Southern blot analysis of *MtAGa* and *MtAGb*. The length of the DNA markers (in Kb) is shown on the left margin. (B) *MtAGb* sequence is anchored in BAC AC153460, on the chromosome 8. Pseudochromosomes are represented by the succession of BACs (vertical, grey thick lines) separated by gaps (black, horizontal lines). Not all gaps are visible at this resolution. Unmapped BACs are shown on the right as MtChr0. MPM1/2: molecular weight markers.
(TIF)

Figure S3 **Molecular characterization of** ***Tnt1*** **insertion line in the** ***mtagb*** **locus.** Genotyping of 20 plants from the *Tnt1* insertion population of NF4908 line. (A) PCR results using AGb-F/Tnt1-F primers. (B) PCR results using AGb-F/AGb-R genomic primers. Homozygous mutant plants are indicated with bold. numbers. MPM: molecular weight markers; WT: wild-type control; H_2_O: distilled water.
(TIF)

Figure S4 **qRT-PCR expression analyses of loss-of-function plants.** Relative expression of *MtAGa* or *MtAGb* genes in flower buds of *mtaga* and *mtagb* mutants. The height of the bars for a given gene indicates differences in relative expression levels in floral buds. The wild-type expression value was set to 1.00, and lower values are plotted relative to this value.
(TIF)

Figure S5 **Floral phenotype and expression analyses of 35S::RNAi-** ***MtAG*** **plants.** (A) Wild-type *M. truncatula* flower. (B-C) Flowers from 35S::RNAi-*MtAG* transgenic line 5.7. (D) Wild-type stamens. (E-F) RNAi lines showing unfused staminal tubes and mild homeotic transformations (arrow). (G) Wild-type carpel. (H-I) Multiple unfused carpels showing exposed ovules (arrow). (J) Relative expression of *MtAGa* or *MtAGb* genes in flower buds of the three transgenic RNAi lines measured by qRT-PCR. The height of the bars for a given gene indicates differences in relative expression levels in floral buds. The wild-type expression value was set to 1.00, and lower values are plotted relative to this value.
(TIF)

Table S1 **Sequences of the C-lineage MADS-box genes from the different species used in the phylogenetic analysis.** The GenBank accession numbers are indicated.
(DOCX)

Table S2 **Primers used in this work.**
(DOCX)
