# Supplementary figures and images for: Two euAGAMOUS Genes Control C-Function in Medicago truncatula
Source: PLoS One. 2014 Aug 8;9(8):e103770. doi: 10.1371/journal.pone.0103770 (PMC4126672; doi:10.1371/journal.pone.0103770)

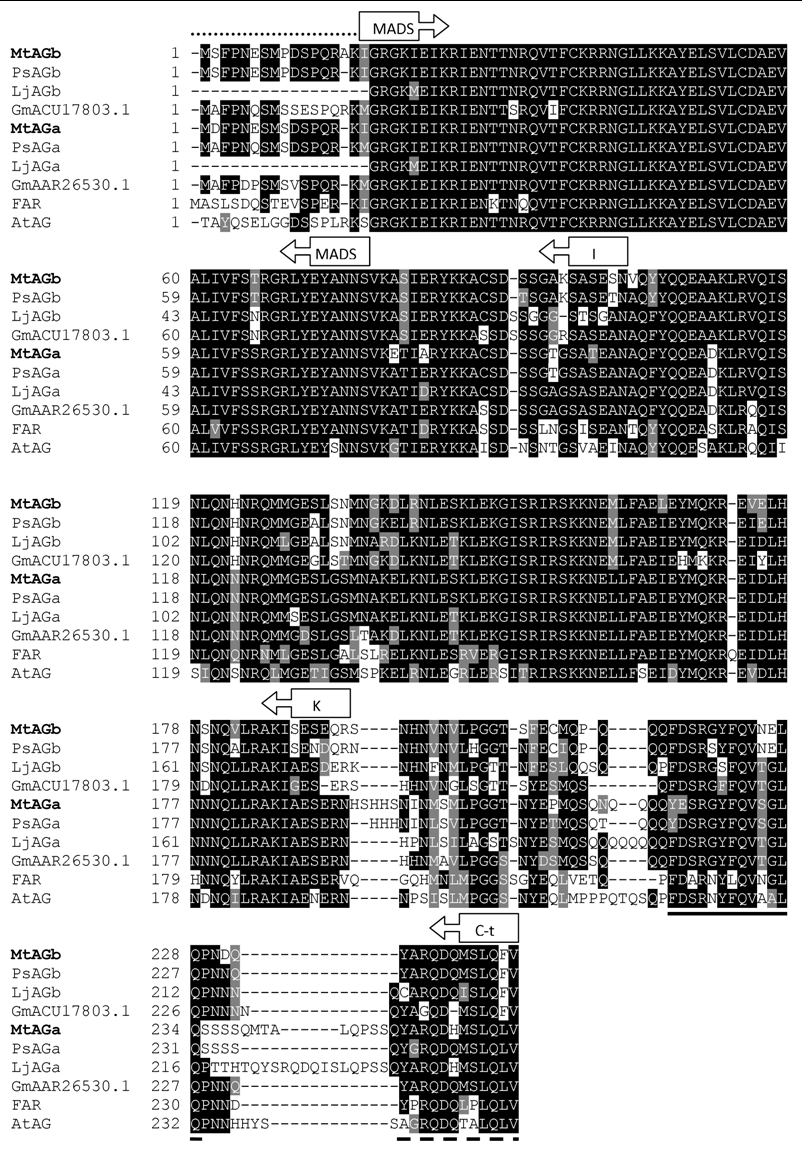

Supplement: Figure S1 — Sequence comparison of MtAGa, MtAGb and related MADS-box proteins. Identical amino acid residues are shaded in black, and similar amino acid residues in grey. The MADS and K domains, and I and C-terminal regions are marked with arrows. The AG motifs I and II are underlined with continuous and discontinuous lines, respectively. A dotted line before the MADS-box indicates the N-terminal sequence characteristic to some C-lineage genes. (TIF) [file pone.0103770.s001.tif]

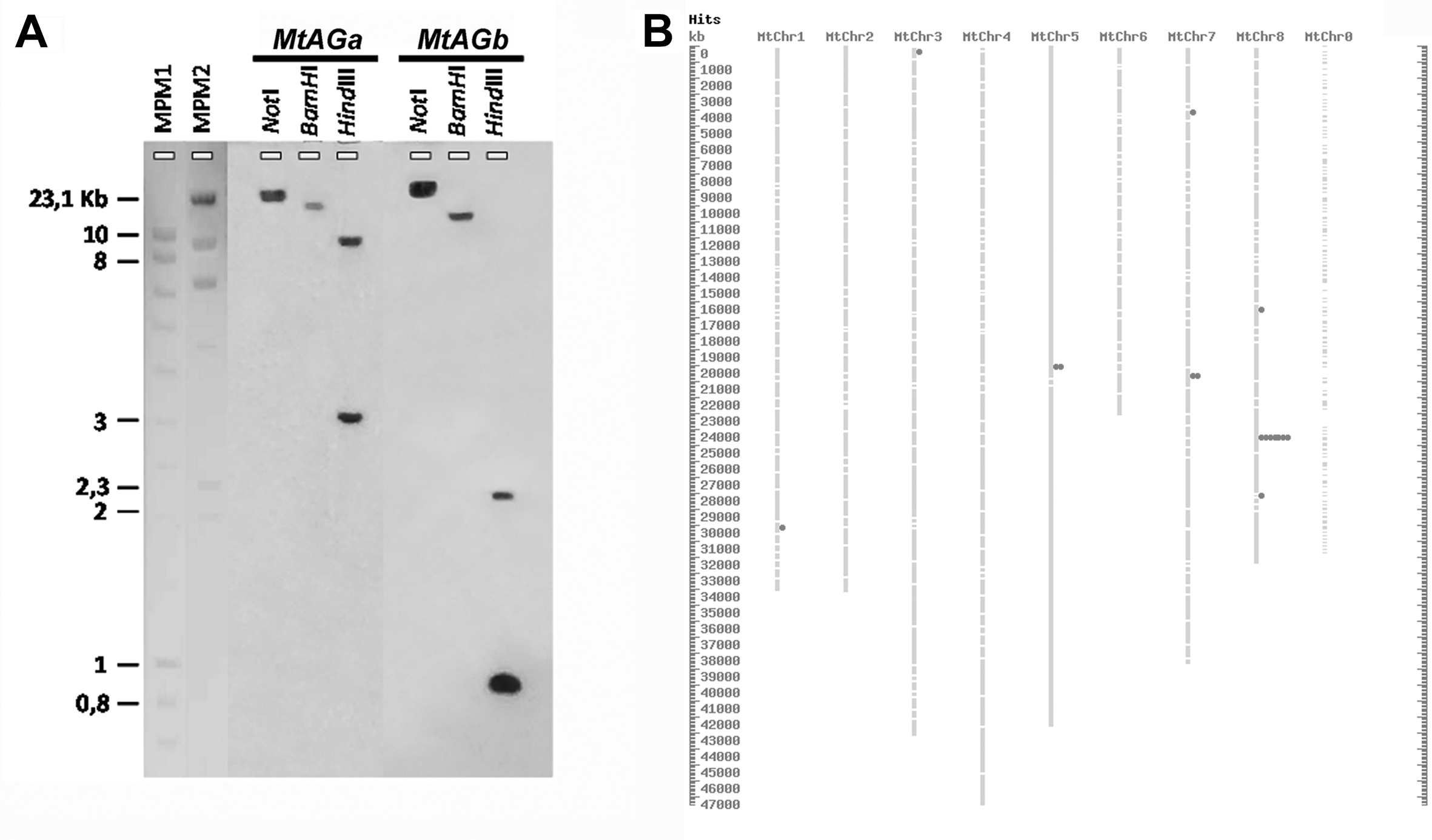

Supplement: Figure S2 — Southern blot analysis of MtAGa and MtAGb genes and localization of the MtAGb gene on the M. truncatula physical map. (A) Southern blot analysis of MtAGa and MtAGb. The length of the DNA markers (in Kb) is shown on the left margin. (B) MtAGb sequence is anchored in BAC AC153460, on the chromosome 8. Pseudochromosomes are represented by the succession of BACs (vertical, grey thick lines) separated by gaps (black, horizontal lines). Not all gaps are visible at this resolution. Unmapped BACs are shown on the right as MtChr0. MPM1/2: molecular weight markers. (TIF) [file pone.0103770.s002.tif]

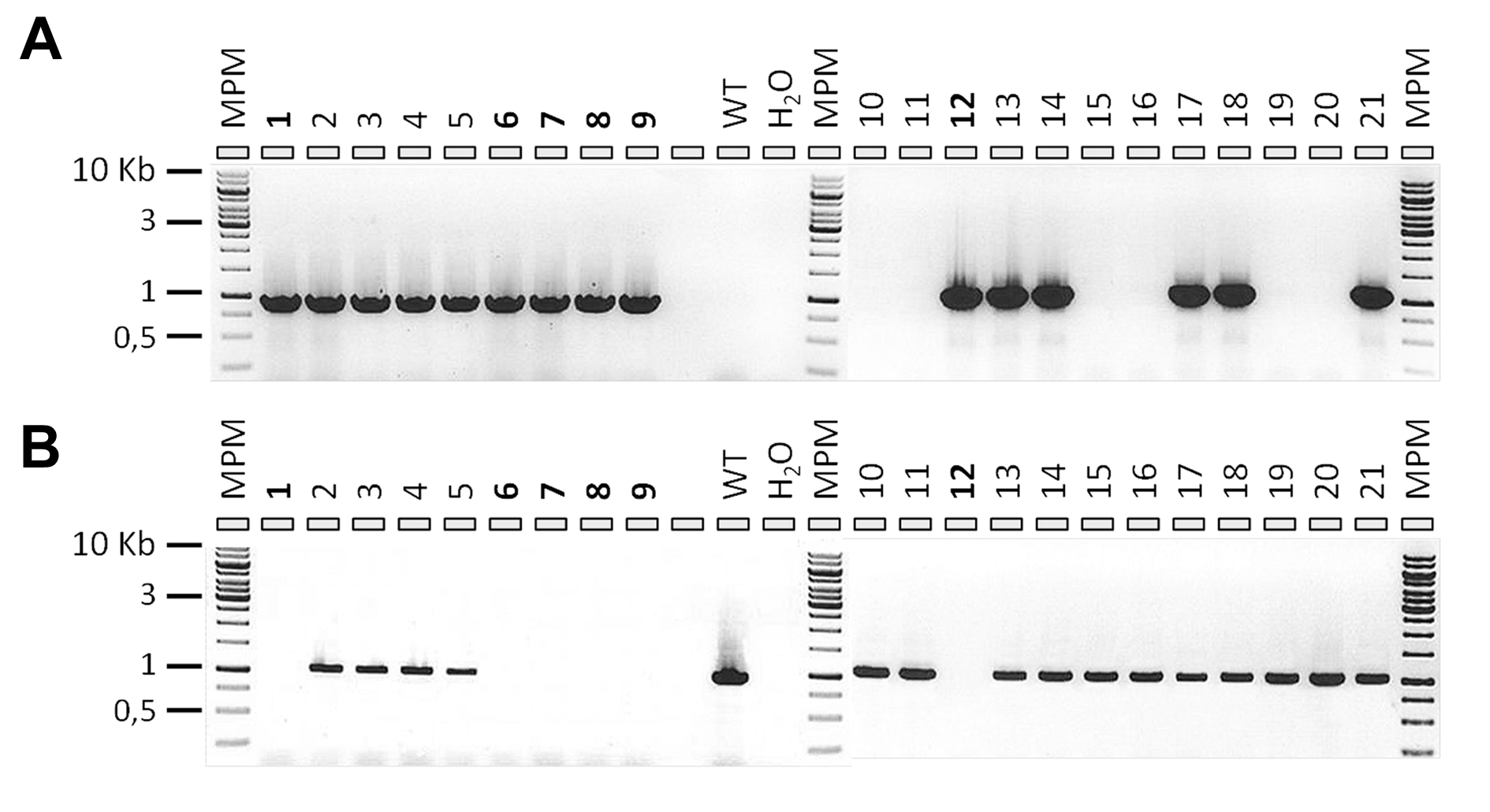

Supplement: Figure S3 — Molecular characterization of Tnt1 insertion line in the mtagb locus. Genotyping of 20 plants from the Tnt1 insertion population of NF4908 line. (A) PCR results using AGb-F/Tnt1-F primers. (B) PCR results using AGb-F/AGb-R genomic primers. Homozygous mutant plants are indicated with bold. numbers. MPM: molecular weight markers; WT: wild-type control; H2O: distilled water. (TIF) [file pone.0103770.s003.tif]

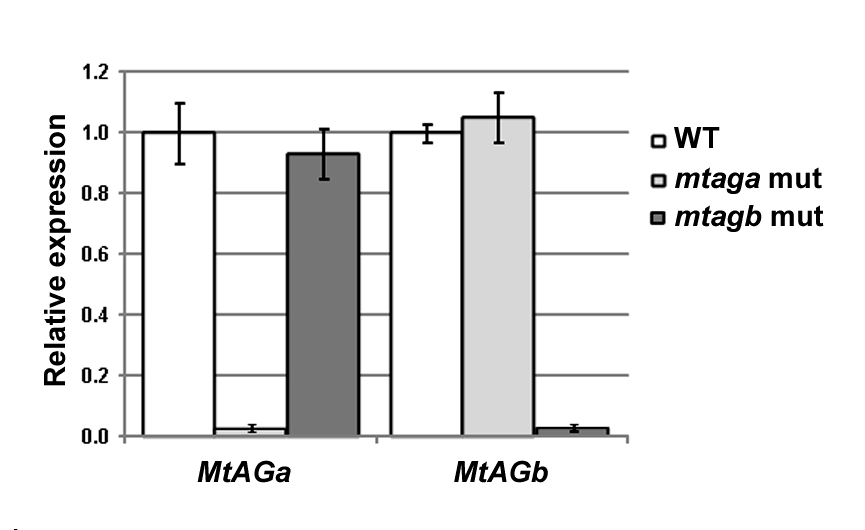

Supplement: Figure S4 — qRT-PCR expression analyses of loss-of-function plants. Relative expression of MtAGa or MtAGb genes in flower buds of mtaga and mtagb mutants. The height of the bars for a given gene indicates differences in relative expression levels in floral buds. The wild-type expression value was set to 1.00, and lower values are plotted relative to this value. (TIF) [file pone.0103770.s004.tif]

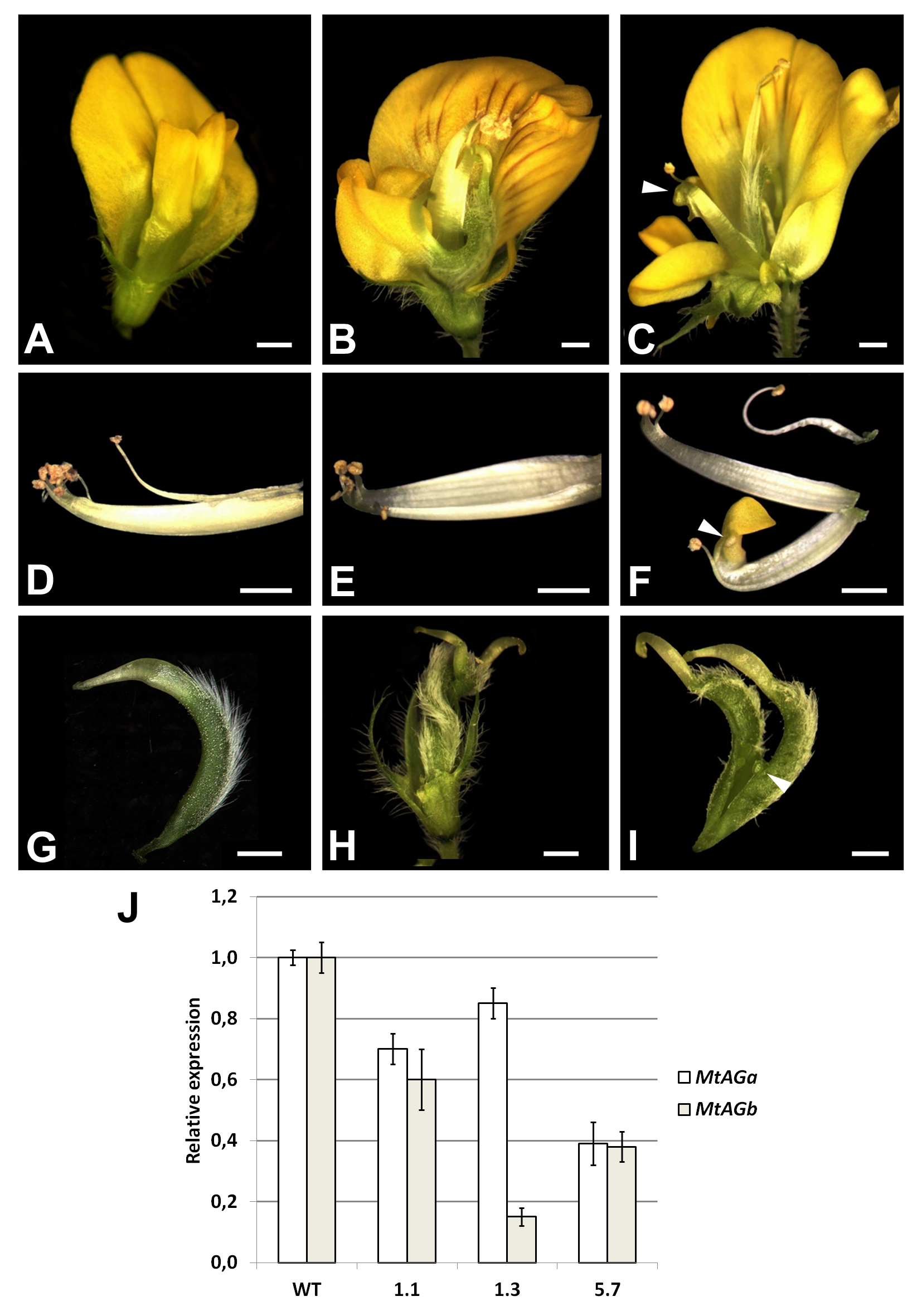

Supplement: Figure S5 — Floral phenotype and expression analyses of 35S::RNAi- MtAG plants. (A) Wild-type M. truncatula flower. (B-C) Flowers from 35S::RNAi-MtAG transgenic line 5.7. (D) Wild-type stamens. (E-F) RNAi lines showing unfused staminal tubes and mild homeotic transformations (arrow). (G) Wild-type carpel. (H-I) Multiple unfused carpels showing exposed ovules (arrow). (J) Relative expression of MtAGa or MtAGb genes in flower buds of the three transgenic RNAi lines measured by qRT-PCR. The height of the bars for a given gene indicates differences in relative expression levels in floral buds. The wild-type expression value was set to 1.00, and lower values are plotted relative to this value. (TIF) [file pone.0103770.s005.tif]
