# Supplementary material for: Two euAGAMOUS Genes Control C-Function in Medicago truncatula
Source: PLoS One. 2014 Aug 8;9(8):e103770. doi: 10.1371/journal.pone.0103770 (PMC4126672; doi:10.1371/journal.pone.0103770)
Supplement: Table S1 — Sequences of the C-lineage MADS-box genes from the different species used in the phylogenetic analysis. The GenBank accession numbers are indicated. (DOCX) [file pone.0103770.s006.docx]

| **Gene name and species** | **Accession numbers** |
| --- | --- |
| *PsAGa* (*Pisum sativum*) | KF147912 |
| *MtAGa* *(Medicago truncatula*) | KF159804 |
| *LjAGa* (*Lotus conicularis japonicus*) | AY770402 |
| *GmAG1* (*Glycine max.)* | AY382833 |
| *GmAG2* (*Glycine max.)* | L0C100776263 |
| *LjAGb* (*Lotus conicularis japonicus*) | AY770403 |
| *PsAGb* (*Pisum sativum*) | AY884291 |
| *MtAGb* (*Medicago truncatula*) | KF159805 |
| *FAR* (*Antirrhinum majus*) | AJ239057 |
| *TAG1* (*Solanum lycopersicum*) | L26295 |
| *PMADS3* (*Petunia hybrida*) | X72912 |
| *NbAG* (*Nicotiana benthamiana*) | JQ699177 |
| *AG* (*Arabidopsis thaliana*) | AT4G18960 |
| *FBP6* (*Petunia hybrida*) | X68675 |
| *NbSHP*(*Nicotiana benthamiana*) | JQ699178 |
| *TAGL1*(*Solanum lycopersicum*) | AY098735 |
| *PLE* (*Antirrhinum majus*) | S53900 |
| *SHP1* (*Arabidopsis thaliana)* | AT3G58780 |
| *SHP2* (*Arabidopsis thaliana)* | AT2G42830 |
| *LjSHP* (*Lotus conicularis japonicus*) | BT135371 |
| *GmAG3* (*Glycine max.)* | L0C100808734 |
| *PsSHP* (*Pisum sativum*) | AY884292 |
| *MtSHP* (*Medicago truncatula*) | JX308825 |
| *EScaAG1* (*Eschscholzia californica*) | DQ088996 |
| *EScaAG2* (*Eschscholzia californica*) | DQ088997 |
| *OsMADS3* (*Oryza sativa*) | L37528 |
| *OsMADS58* (*Oryza sativa)* | AB232157 |
| *STK* (*Arabidopsis thaliana*) | AT4G09960 |
