# Supplementary material for: Two euAGAMOUS Genes Control C-Function in Medicago truncatula
Source: PLoS One. 2014 Aug 8;9(8):e103770. doi: 10.1371/journal.pone.0103770 (PMC4126672; doi:10.1371/journal.pone.0103770)
Supplement: Table S2 — Primers used in this work. (DOCX) [file pone.0103770.s007.docx]

| **Primers** | **Sequences 5’-3’** |
| --- | --- |
| MtAGb-F | TCCAAACGAATCCATGCCAGATTC |
| MtAGb-Rgenomic | GTTTATCCAAGCTGAGATTTGAGG |
| Tnt1-F | ACAGTGCTACCTCCTCTGGATG |
| Tnt1-F1 | TCCTTGTTGGATTGGTAGCCAACTTTGTTG |
| Tnt1-R | CAGTGAACGAGCAGAACCTGTG |
| Tnt1-R1 | TGTAGCACCGAGATACGGTAATTAACA |
| MtAGa-intron-F | TGGAAGGGGAAAGATTGAGA |
| MtAGa-intron-R | CAGATCCAGTGCCAGATGAA |
| MtSHP-intron-F | GAAGGTGGAGAAGGATCTTCTCAAAAG |
| MtSHP-intron-R | CTGCTGATCTGATCCAAGAAGATTTAC |
| MtAGadir | GGAACCATAGCCACCATAGC |
| MtAGarev | CTTTCATACTTCTCTCAAGCAC |
| MtAGbdir | GATATCAGAAAGTGAGCAGAG |
| MtAGbrev1 | GTCTTTGCTCTTCTCAACCG |
| MtAGa-qPCRdir | AGAGCAAAGATAGCTGAAAGTGAGAG |
| MtAGa-qPCRrev | ATTAATATTGCTATGGTGGCTATGG |
| MtAGb-qPCRdir | ACATTGAGTATGGAGATTACACATTGG |
| MtAGb-qPCRrev | GAAAGTAGAATAAGGGTGATACATGCC |
| TC77416dir | TGGCTACTAGGGTTGCTGGC |
| TC77416rev | CCTCACCCAGTCCAGTGGAA |
| MtAGaVIGSdir | GCCATGGAACCATAGCCACCATAG |
| MtAGbVIGSdir | GTCTAGAAAGTGAGCAGAGGAGCA |
| MtAGaVIGS2rev | CTCTAGACTTAGCACTCATCTGCTATC |
| MtAGbVIGS2rev | CCTGCAGATACAAAGCCACTACATAG |
| MtAGb-RNAiD | CTCGAGTCTAGAGATATCAGAAAGTGAGCAGAG |
| MtAGb-RNAiR | GAATTCGGATCCGTCTTTGCTCTTCTCAACCG |
| AGaSBXdir | TGTCGACATGGATTTTCCAAATGAATCCAT |
| AGaSBXrev | TGGATCCTCAAACTAATTGAAGGGACATG |
| AGbSBXdir | TGTCGACATGAGTTTTCCAAACGAATCC |
| AGbSBXrev | TGGATCCTTACACAAATTGAAGAGACATC |
